# Epidemiology of Dementia among the Elderly in Sub-Saharan Africa

**DOI:** 10.1155/2014/195750

**Published:** 2014-08-06

**Authors:** Olaniyi O. Olayinka, Nadine N. Mbuyi

**Affiliations:** ^1^Department of Neurology and Psychiatry and Department of Environmental and Occupational Health, Saint Louis University, Saint Louis, MO 63104, USA; ^2^Department of Internal Medicine, Roger Williams Medical Center, Boston University School of Medicine, 825 Chalkstone Avenue, Providence, RI 02908, USA

## Abstract

*Objectives.* To review epidemiologic studies on the prevalence, incidence, and risk factors of dementia in sub-Saharan Africa (SSA). *Methods.* A MEDLINE search (from January 1992 to December 31, 2013) of epidemiologic studies, with no language restriction, was conducted using the keywords “dementia” or “Alzheimer's” and “Africa.” We selected for review population and hospital-based studies that reported the prevalence, incidence, or risk factors of dementia in SSA in people aged 60 years and above. References of selected articles were reviewed to identify additional relevant articles that met our selection criteria. *Results.* Of a total of 522 articles, 41 were selected and reviewed. The reported prevalence of dementia in SSA varied widely (range: 2.29%–21.60%); Alzheimer's disease was the most prevalent type of dementia. Only two studies conducted in Nigeria reported incidence data. Major risk factors identified include older age, female gender, cardiovascular disease, and illiteracy. *Conclusion.* Data on the epidemiology of dementia in SSA is limited. While earlier studies reported a lower prevalence of dementia in older persons, recent studies have put these findings into question suggesting that dementia prevalence rates in SSA in fact parallel data from Western countries.

## 1. Introduction

According to a recent survey conducted by Harvard School of Public Health and Alzheimer's Europe consortium on Alzheimer's disease (AD), dementia of the Alzheimer's type is a major cause of health concern in adults [1]. A similar trend is predicted in most countries in sub-Saharan Africa (SSA) where the proportion of elderly people is expected to rise over the coming years [2]. Worldwide, the prevalence of dementia among those aged 60 years and above ranges from five to seven percent [3] and studies conducted in developed countries have consistently shown that AD is the most common type of dementia followed by vascular dementia (VaD) [4]. However, there are scarce and conflicting reports on the prevalence of dementia and its subtypes in SSA [3, 5, 6], which may have far-reaching implications on public health policies on dementia in the region. While some studies suggest a lower prevalence of dementia in some parts of SSA [3, 7–10], other studies report prevalence rates comparable to those reported from Western countries [6, 11, 12].

A variety of reasons may account for the varying reported prevalence rates of dementia in SSA. This is a vast territory with a population of 1.1 billion people [13, 14], and the number of people aged 60 years and above is projected to rise to over 67 million by 2030 [15]. The region consists of several ethnic groups, with different cultures, languages, diets, and traditions [13], which may impact the prevalence of diseases in unique ways. Additionally, the relatively high prevalence of communicable diseases such as HIV-AIDS, low life expectancy at birth, the rising prevalence of cardiovascular risk factors such as hypertension and diabetes, and low-literacy level in SSA are important factors that are also reported to influence the epidemiology of chronic diseases like dementia [16, 17]. Further, genes play a role in the development of dementia and may impact its epidemiology in the genetically diverse African population [18]. The factors mentioned above, combined with the limited amount of region-wide, standardized epidemiological studies in SSA, call for a review of studies on dementia in the region. The objective of this paper is to identify the prevalence, incidence, and risk factors of dementia in SSA in order to gain a clearer view of this major public health concern.

## 2. Methods

**Figure 1:**
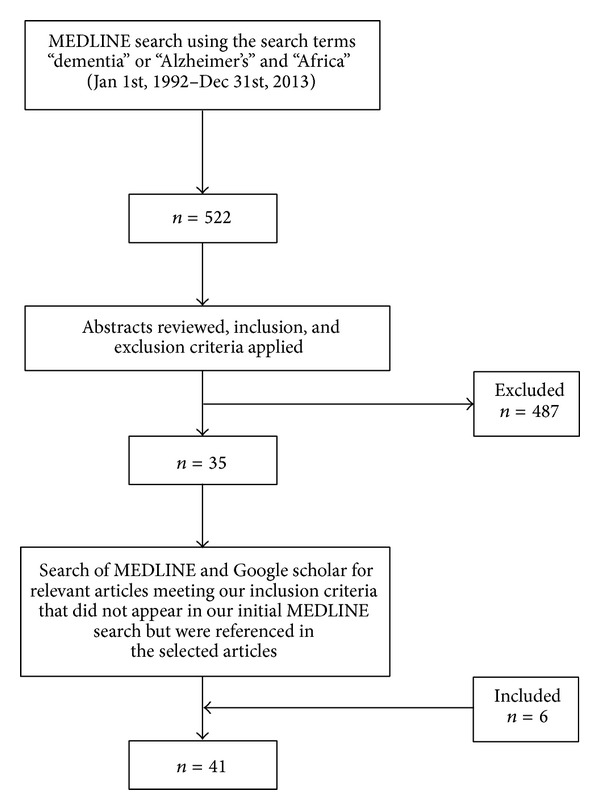
Flow chart of search methodology used to identify relevant population and hospital-based studies on the epidemiology of dementia in SSA.

**Figure 2:**
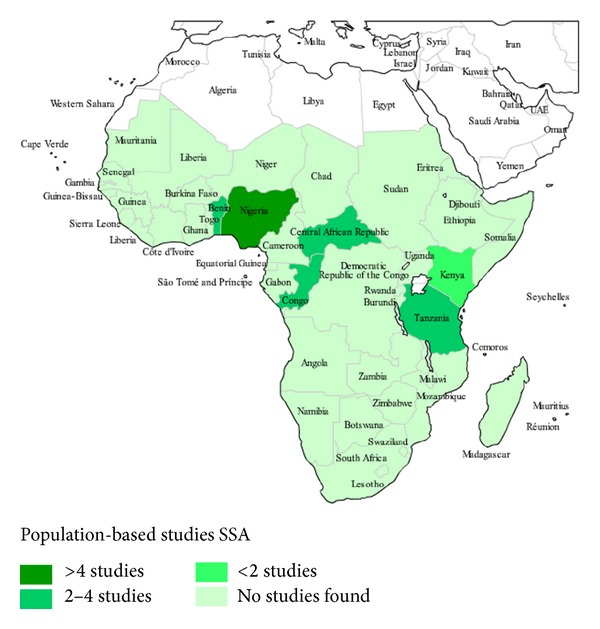
Distribution of population-based studies on the epidemiology of dementia in SSA.

We conducted a search of MEDLINE (from January 1st, 1992 to December 31, 2013) for epidemiologic studies using the keywords “dementia” or “Alzheimer's” and “Africa,” with no restriction to language (Figure 1). Subsequently, each author reviewed the abstracts of all articles that were identified through the search. Studies were selected for further review and analysis if they satisfied the following inclusion criteria:

1. studies that related epidemiologic data (i.e., prevalence, incidence, or risk factors) on dementia in SSA (i.e., 49 sovereign nations, 2 overseas departments of France, and 1 overseas British territory located fully or partially south of the Sahara desert) [13] (Figure 2);
2. studies that were population or hospital based;
3. studies that included persons aged 60 years and above;
4. studies that used validated screening and diagnostic tools for dementia and dementia subtypes;
5. studies describing histological findings from brain autopsies of elderly subjects.

References of selected articles were also reviewed in order to identify additional relevant articles that met our selection criteria. When there was a disagreement between the authors as to whether an article should be selected for review, a discussion took place and both authors reached a consensus.

Studies were excluded on the basis of the following exclusion criteria:

1. studies not conducted in SSA;
2. studies that were reviews, case reports, or case series;
3. studies that did not relate epidemiologic data;
4. studies that did not discuss dementia or its subtypes in individuals aged 60 years and above.

A summary of the studies that were selected is provided in Tables 1 and 2.

## 3. Results

The search yielded 522 articles. The abstracts of all the articles were reviewed and 35, that met the inclusion criteria, were selected for further review (Figure 1). Six additional articles were identified from the references of the selected articles, making a total of 41 articles that were finally reviewed. The 41 articles included one study (2.44%) each from Burkina Faso, Cameroon, Ghana, and the Republic of Congo, two studies (4.88%) each from Benin Republic, Kenya, Senegal, and South Africa, three studies (7.32%) from Central African Republic and Tanzania, and 23 studies (56.10%) from Nigeria. Twenty-seven (65.85%) studies were population-based; thirteen (31.71%) were hospital-based studies. Most of the population-based studies had study participants ranging from 184 persons in a Kenyan study (84 controls versus 100 demented persons) to 4,706 persons in the Indianapolis-Ibadan epidemiological study of dementia (2,494 Nigeria-Yoruba versus 2,212 African Americans in Indianapolis), while the range for hospital-based studies was 23 to 240,294 participants (Tables 1 and 2).

Overall, the reported age-adjusted prevalence of dementia for the population-based studies we reviewed varied widely ranging from 2.29% (AD = 1.41%) in Nigeria-Yoruba, aged 65 years and above, who lived in the Idikan community in Ibadan city [8], to 21.60% (AD prevalence not reported) in the rural Hai district of Tanzania (study participants aged 70 years and above) [6]. The reported prevalence of dementia for hospital-based studies ranged from 0.05% at a neuropsychiatric practice in southwestern Nigeria (survey period 1998–2007) [45], to 8.87% at a geriatric center in Dakar, Senegal [51]. Further, dementia accounted for 6.90% of patients with acute confusional state in a hospital in Tanzania [48], while 74.00% of 305 patients who presented to a memory clinic in South Africa were diagnosed with dementia [53]. The incidence of dementia in SSA was only reported in two population-based studies both conducted in Nigeria-Yorubas. The age-standardized annual incidence rate of dementia reported by one of the studies was 1.35% (AD = 1.15%) [48], while the other study reported a dementia incidence of 2.19% in a survey of 1225 Yoruba subjects, aged 65 years and above [24]. Major risk factors for dementia that were identified by most of the population-based studies included increasing age, female gender, cardiovascular disease, and low-literacy level [6, 10, 35]. In most studies, AD was the most reported dementia subtype [8–10, 22, 36, 52]. While there were reports of a high frequency of the APOE-*ɛ*4 allele in Africans, most studies found no significant association between the APOE-*ɛ*4 allele and dementia in SSA [8, 9].

### 3.1. Prevalence

The first community-based study we found on the prevalence of dementia in SSA was conducted in Ibadan, Nigeria, in 1992. The authors found impaired cognition in 4.4% of the 932 study participants (293 were aged 65 years and above), and none met the DSM-III-R diagnostic criteria for dementia, suggesting that dementia was a rare disease in Nigeria [19]. A year later, the authors conducted a similar but hospital-based study in which they found that 37 out of a total of 57,440 hospitalized patients had dementia [41]. Of the 37 patients, 18 (48.70%) had vascular dementia and one (2.70%) patient was identified as having probable primary neurodegenerative disease. Additionally, a study of 198 postmortem brains conducted at a university hospital in Ibadan revealed a paucity of histological markers of AD (cortical neuronal loss, amyloid beta plaques, neurofibrillary tangles, and amyloid angiopathy) [42].

In 1995, prevalence data on dementia from a collaborative project between researchers in Indianapolis, United States, and Ibadan, Nigeria, the Indianapolis Ibadan Dementia Research Project (IIDP), was reported. The authors found the prevalence of dementia in an Ibadan community to be 2.29% (AD = 1.41%) [9]. The study enrolled 2494 Yorubas and 2212 African American Indianapolis residents, aged 65 years and above. It is worth noting that the dementia screening instruments used for the Ibadan-Indianapolis study was customized to reflect the literacy level and culture of the study participants. In another well-powered, cross-sectional survey of persons who lived in the rural Djidja community of Benin, the reported prevalence of dementia was also low at 2.60% in those aged 65 years and above [9]. Two years later, a similar prevalence study conducted in the urban city of Cotonou in Benin, reported a nonsignificantly higher but comparable prevalence of 3.70% [10]. A lower prevalence of 2.85% was also reported at a neurology clinic in Yaoundé, the capital of Cameroon, where the authors reviewed the medical records of 912 patients who visited the clinic from May 2005 to December 2011 [55]. The Cameroon finding is similar to a study conducted in eastern Nigeria where dementia accounted for 3.00% of all neurological admissions into the medical ward of a university hospital between 2003 and 2007 [44]. In northern Nigeria, the prevalence of dementia in a cross-sectional survey of 322 Hausa-Fulanis, aged 65 years and above, was 2.79% with AD accounting for 66.67% of cases [7].

Higher prevalence of dementia has also been reported in parts of SSA. Although this was a survey of nursing-home residents in cosmopolitan Lagos, the authors found that 11 (37.93%) of the 29 residents had dementia, a prevalence that is close to those reported in similar institutions in Western countries [43]. Further, Turkson and Asamoah reported that dementia was the second most common diagnosis made at a psychiatric out-patient clinic in Accra, Ghana, between 1989 and 1993 in 35 patients, aged 60 years and above [46]. Additionally, a cross-sectional survey conducted in central Nigeria reported a slightly higher prevalence of dementia (6.40%) [33] than was reported in studies conducted in Ibadan, Nigeria [8]. Two hospital-based surveys in Senegal equally reported higher prevalence rates of dementia of 6.60% (in participants aged 55 years and above) and 8.87% (participants aged 65 years and above) [49, 51]. Similarly, a population-based study conducted in the Central African Republic capital city of Bangui reported a dementia prevalence of 8.10%, a rate that is similar to data from Western countries [12]. In 2012/13, a two-phase cross-sectional survey of six villages was conducted in the rural Hai district of Tanzania (subjects aged 70 years and above) using two different diagnostic tools for dementia, the DSM-IV and the 10/66 Dementia Research Group diagnostic criteria for dementia for low and middle income countries [6, 40]. Surprisingly, a higher dementia prevalence of 21.60% was found using the 10/66 diagnostic criteria compared to a prevalence rate of 6.40% when diagnosis was based on the DSM-IV criteria [6, 40].

### 3.2. Incidence

Only two of the studies we reviewed reported incidence data on dementia, and both were population-based longitudinal studies conducted in Ibadan, Nigeria [24, 35]. After a five-year followup, the first study reported the age-adjusted incidence of dementia in persons aged 65 years and above as 1.35% (AD = 1.15%) [24], while the estimated incidence of dementia in a similar cohort of elderly participants in a 2011 study was 2.19% after a 3-year followup [35].

### 3.3. Risk Factors

A fairly broad range of genetic and environmental risk factors for dementia was reported by many of the studies we reviewed. Studies conducted in Nigeria, Kenya, Tanzania, and Benin all reported a high frequency of the APOE-*ɛ*4 allele (genetic risk factor for AD) in Africa [8, 9, 20, 25, 26, 39]. Despite the high frequency of the APOE-*ɛ*4 allele found in Africans, there was a general lack of association between the allele and dementia in the region [21, 25, 39]. Interestingly, Guerchet et al., in their Benin study, reported a significantly lower frequency of the APOE-*ɛ*2 allele in study participants with dementia [9].

Interaction between genetic and environmental risk factors in the development of AD was highlighted in some studies. For instance, Hall et al. found that in Yoruba subjects who had high levels of cholesterol and LDL in conjunction with the APOE-*ɛ*4 allele had a decreased risk of AD compared to subjects without the allele [26]. The effect of gene-gene interaction on dementia was reported by a study that aimed to compare the age- and gender-specific distribution of the APOE and alpha-1-antichymotrypsin (ACT) genes and the effect of this interaction on AD [20]. In this study, Kamboh et al. suggested that the higher prevalence of AD in both Caucasian and Black women may be due to the modifying effects that ACT has on the APOE gene. Specifically, the study found a higher frequency of the ACT^∗^A and APOE 4 alleles in Nigerian Blacks. Furthermore, these two alleles were found to occur “nonrandomly” in both Caucasian and Nigerian women. The authors hypothesized that the interaction between these two alleles may account for the higher prevalence of dementia observed in women [20].

Environmental factors reported to be associated with dementia in SSA include the following: increasing age, female sex, positive history of hypertension, hypercholesterolemia, diabetes mellitus, peripheral arterial disease, stroke, low level of education, diet, depressive symptoms, and low body mass index [8, 9, 27, 29, 32, 33, 35, 37, 38, 47, 54]. In an IIDP study, a comparison of smoking history and mean body mass index (BMI) showed that these factors were significantly higher in African-Americans than in the Yoruba suggesting that environmental factors play a significant role in the development of Alzheimer's dementia [23]. An additional two studies found hypertension and low BMI to be associated with an increased risk of dementia in elderly Yoruba [30, 31]. Similarly, increasing age, female gender, hypertension, low weight, living alone, and low education level were reported to be significantly associated with dementia in a survey of elderly study participants living in Bangui, Central African Republic and Brazzaville, Congo [37]. It is worth noting that Ogunniyi et al. found that self-reported hypertension was a protective factor for Alzheimer's dementia in Yorubas [27]. Increased homocysteine levels were also reported to be associated with a similar but nonsignificant increase in dementia risk for both Yoruba and African Americans although there was a significant difference in folate levels between the two sites [32]. Further, two Senegal studies linked age, illiteracy, and low social network to the development of dementia [49, 51]. The Senegal findings are consistent with those of a cross-sectional study conducted in South Africa. The South Africa study found that participants with dementia and mild cognitive impairment were of advanced age and had less than 12 years of formal education in addition to having a higher prevalence of vascular events such as hypertension and stroke. However, this study found no significant association between the sex of participants and dementia [54]. More recently personality change was reported to be an indicator for future dementia in elderly African Americans and Nigeria-Yoruba [28].

## 4. Discussion

This review highlights the fact that the reported prevalence of dementia in SSA varies widely [56]. However, most studies suggest a lower prevalence of dementia compared to developed countries. Because age is the strongest risk factor for dementia [57, 58], the low prevalence of dementia reported in SSA may be associated with the low life expectancy in the region. Additionally, the paucity of epidemiologic data on dementia makes it difficult to get an overall picture of the burden of the disease in this region. Research methodology may also affect epidemiologic data [56]. For instance, some of the articles we reviewed were self-reported surveys. Employing this approach might contribute to the reported low prevalence of dementia because, in several African cultures, people with dementia and other mental health problems are often stigmatized [59]. Furthermore, only few studies provided age-adjusted rates for dementia despite differences in the age-structure of countries in SSA [15]. Therefore, inferences based on a comparison of crude prevalence data on dementia across studies may be misleading. For hospital-based studies, low utilization of healthcare facilities by the elderly might have contributed to the reported low prevalence rates of dementia.

Various cultures and languages exist within and between countries in SSA. This may also pose a special challenge for researchers working on dementia who adopt standardized dementia screening tools that were originally developed for Western societies [60]. Most of the studies included in our review used standardized screening tools for dementia in accordance with internationally recognized guidelines. However, some researchers customized these screening tools to the culture, language, and literacy level of study participants, as was the case with the IIDP and the “test of Senegal,” a dementia-screening tool [50], among other studies [6, 8, 34, 35]. This approach is novel and could be considered in developing a harmonized screening tool for dementia in SSA in the future.

Similarly, identifying risk factors for dementia in SSA is critical. The finding that there was a lack of association between the APOE-*ɛ*4 allele and dementia appears to be unique to the African population as the presence of this allele has been associated with a higher prevalence of AD in Caucasians and African Americans [25, 39]. In fact, Hall et al. found a higher risk for AD in subject participants with high cholesterol and LDL levels who lack the APOE-*ɛ*4 allele [26]. Additionally, the finding that the absence of the APOE-*ɛ*2 allele in study participants in Benin is significantly associated with dementia, and cognitive impairment is unique and calls attention to the role that genes play in the prevalence of dementia in specific SSA populations [9]. Further, urban versus rural living was found to be associated with dementia. While two studies conducted in Benin found a nonsignificantly lower prevalence of dementia in the rural Djidja community (2.60%) compared to the urban city of Cotonou, Benin, (3.70%) [9, 10], Gureje et al. [35] found rural living to be associated with an increasing incidence of dementia in southwestern Nigeria [35]. Correlations between different cardiovascular risk factors and dementia were also reported in some studies. For instance, Guerchet et al. attributed the higher prevalence of dementia in two urban cities of central Africa to hypertension, among other risk factors [37]. The lower prevalence of cardiovascular diseases and diabetes in Yoruba compared to African Americans living in Indianapolis was also suggested to play a role in the low prevalence of dementia in Yorubas [23]. The Yoruba diet which is low in calories, fat, and salt but high in fiber and ascorbic acid was also identified as being protective against AD [61]. Overall, the studies reviewed suggest that genetic and environmental factors also affect the epidemiology of dementia in SSA. However, further research into the association between these factors and dementia should be encouraged.

### 4.1. Strengths and Limitations

This review provides an overview of the scope of epidemiologic studies conducted on dementia in SSA. To that effect, both community and hospital-based studies conducted over the last twenty-two years were included in our analysis. In addition to presenting prevalence data, we also reviewed available data on the incidence and risk factors (including genetic and environmental factors) of dementia in SSA. However, we identified some limitations. For instance, our initial search was limited to the MEDLINE database and might have excluded some relevant work. We addressed this issue by reviewing references of articles identified through our initial search. Finally, because most of the studies we found were conducted in single communities in various countries across SSA, the results lack external validity.

## 5. Conclusion

This review of published work on the epidemiology of dementia in older persons in SSA suggests that research in this area is limited. Most of the population-based studies were in single communities and may not be representative of the country in which they were conducted. Overall, earlier studies reported a lower prevalence of dementia, while recent studies have put these findings into question suggesting that dementia prevalence rates in SSA may in fact be similar to Western countries. Based on these observations, more epidemiologic studies that are representative of the SSA population should be encouraged. This may involve collaborative projects between researchers within and between SSA countries. For instance, comparison of genetic polymorphisms between different communities in the region may unravel subtle factors that contribute to the epidemiology of dementia. The use of a harmonized screening tool for dementia that accounts for differences in literacy levels and sociocultural characteristics is also suggested. This would require dementia researchers in SSA to work together. In other regions of the world, dementia has been established as a disease of high economic and public health significance. As the population of the elderly is predicted to rise globally, the burden of dementia in SSA is likely to increase. Therefore, stakeholders in public health in the region are advised to map out strategies to tackle this important public health issue.

## Figures and Tables

**Table 1:** Epidemiologic research on dementia in sub-Saharan Africa—population-based studies.

Country	Authors	Study design/methodology	Prevalence rate	Incidence rate	Risk factors/associated conditions
Nigeria	Ogunniyi et al., **1992** [19]	(i) Community-based, cross-sectional survey in Idikan NW3 ward (ii) *n* = 932 (iii) Age ≥ 40 years; 293 subjects were ≥65 years (iv) Screening and diagnosis of dementia: modified ∗MMSE, ∗DSM-III-R	No prevalence data reported as none of the subjects met diagnostic criteria for dementia per DSM-III-R		Decline in cognitive function significantly correlated with age, female sex, and low level of education
				
	Kamboh et al., **1997** [20]	(i) Population-based study (ii) Aim: to determine prevalence of APOE-*ɛ*4 and association between 1-antichymotrypsin (ACT) and APOE-*ɛ*4 loci and risk of ∗AD in Nigerian Blacks versus Caucasians living in Pittsburgh, USA, and women versus men (iii) *n* = 1,533 (803 Caucasians and 730 Nigerian Blacks) (iv) Age: 40–89 (mean 57.5) for Caucasian and 19–70 (mean 40.8) for Nigerian Blacks	Authors note that despite much higher prevalence of APOE-*ɛ*4 and ACT∗A carriers in Blacks, prevalence of AD in Blacks was found to be comparable to, or even lower than in Caucasians in other studies		(i) Distributions of ACT and APOE alleles are significantly different between Caucasians and Nigerian Blacks (ii) There was a nonrandom association between the two polymorphisms in Caucasian and Nigerian women; this may in part explain higher prevalence of dementia in women versus men
	**Indianapolis Ibadan Dementia Project (IIDP) Articles** Hendrie et al., **1995** [8], Osuntokun et al. **1995b** [21], Ogunniyi et al., **1997** [22], Ogunniyi et al., **2000** [23], Hendrie et al., **2001** [24], Gureje et al., **2006a** [25], Hall et al., **2006** [26], Ogunniyi et al., **2006** [27], Smith-Gamble et al., **2002** [28], Ogunniyi et al., **2011** [29–31], and Hendrie et al., **2013** [32]	Longitudinal prospective comparative study (survey conducted in two phases) **Phase 1: initial selection criteria** (i) Age ≥ 65 years (ii) Community-dwelling elderly African Americans living in Indianapolis and Yoruba living in Ibadan, Nigeria (iii) Screening tool for dementia: harmonized ∗CSID *Outcome* 2494 Yoruba versus 2212 African Americans selected **Phase 2: final selection criteria based on clinical assessment** *Diagnostic tools* (i) Dementia: DMS-III-R and ∗ICD-10 (ii) Severity of dementia: DSM-III-R, ICD-10, and clinical dementia ratings (iii) Probable or possible AD: ∗NINCDS-ADRDA *Outcome * 423 Yoruba versus 351 African Americans included	**1995**: age-adjusted prevalence of **dementia** (**2.29%** versus 4.82%) and **AD** (**1.41%** versus 3.69%) were found to be lower in Yoruba than in ∗AA and, respectively, in community dwelling subjects (i) **1997**: door to door survey in Idikan NW3 ward in subjects ≥65 years contested previous claims by Ogunniyi et al., 1992 [19], that dementia and AD were rare in this community (ii) 28 out of 2,494 subjects screened were found to have dementia. 18 subjects (64.3% of cases) were found to have AD (iii) ∗VaD was the 2nd most common subtype after AD	**2001**: Age-standardized annual incidence rates of **dementia** (**1.35%** versus 3.24%) and **AD** (**1.15%** versus 2.52%) found to be lower in Yoruba than AA	(i) **1995**: in both AA and Yoruba, age was associated with increased dementia prevalence and AD was the most common dementia subtype (ii) Prevalence of APOE-*ɛ*4 allele high among elderly Yoruba but lack of association between the allele and AD among Yoruba subjects versus AA (iii) **1997**: increasing age and female gender were found to be risk factors for AD. Living with others was protective against dementia (iv) **2000–2006**: lack of association between AD and possession of APOE-*ɛ*4 allele in Nigerian sample, unlike AA (v) Cardiovascular risk factors (hypertension, diabetes, stroke, smoking, and mean BMI) were found to be higher in AA than in Yoruba suggesting that environmental factors play a major role in the development of dementia (vi) Increasing levels of cholesterol levels and LDL associated with increasing risk of AD in individuals without the APOE-*ɛ*4 allele but not in those with APOE-*ɛ*4 in Yoruba subjects (vii) Increasing age and female gender associated with increased risk of incident dementia (viii) **2011**: personality change is a significant predictor of future dementia, independent of cognition and functional status, in both Yoruba and AA (ix) Hypertension and decline in BMI are associated with increased risk of incident dementia in elderly Yoruba (x) **2013**: increased homocysteine levels associated with similar but nonsignificant increase in dementia risk for both Yoruba and AA subjects despite significant differences in folate levels between the two regions
	Ochayi and Thacher, **2006** [33]	(i) Cross-sectional survey Nigeria (Jos, Central Nigeria) Age ≥ 65 years (ii) *n* = 280 (iii) Central Nigeria (iv) Diagnostic tool: CSID	Slightly higher prevalence of dementia (6.4%) than reported in older studies from Ibadan in Southern Nigeria (2.29%)		(i) Female sex, low body mass index (BMI), lack of NSAID use, and increasing age are risk factors for dementia (ii) Lack of association between level of education and dementia
	**Ibadan Study of Aging (ISA) articles** Gureje et al., **2006** [34], Gureje et al., **2011** [35]	(i) Community-based survey in 8 Yoruba speaking Nigerian states (ii) *n* = 2152 (iii) Age ≥ 65 years (iv) 3-year followup (v) Dementia assessment: 10-word delayed recall test and clinician home-based interview to assess function	**2006**: Prevalence of probable dementia was 10.1%	**2011**: Estimated incidence of dementia was 21.85 per 1000 person-years	(i) **2006**: female gender and increasing age were risk factors (ii) Lifetime history of alcohol use doubles the risk (iii) **2011**: increasing incidence of dementia in rural areas. Social isolation and low economic status were identified as risk factors for incident dementia
	Yusuf et al., **2011** [7, 36]	(i) Cross-sectional, community-based survey (ii) Nigeria (Zaria), Northern Nigeria (iii) Age ≥= 65 years (iv) *n* = 322, urban area (v) Screening: CSID, ∗CERAD, ∗SDT, Blessed Dementia Scale and a sociodemographic questionnaire (vi) Dementia diagnosis: ∗ICD-10 and DSM-IV	Prevalence of dementia estimated at 2.79% with AD accounting for most cases in the community studied (66.67%)		Age was identified as risk factor for dementia

Benin	Guerchet et al., **2009** [9]	(i) Cross-sectional community-based survey (ii) Rural community versus urban community (iii) Age ≥ 65 years (iv) *n* = 502 (v) Screening: CSID and five-word test (vi) Dementia diagnosis: DSM-IV (vii) Probable and possible AD: NINCDS-ADRDA	(i) Prevalence of dementia is 2.6% in this rural community (ii) Dementia prevalence appears to be lower in developed countries (iii) AD is the most common cause of dementia rural setting		(i) Low prevalence of dementia in this rural community despite high a high prevalence of the APOE-*ɛ*4 allele in study participants (ii) There was a significantly lower frequency of the APOE-*ɛ*2 allele in study participants with dementia
	Paraïso et al., **2011** [10]	(i) Cross-sectional community-based study (ii) Urban community (iii) Age ≥ 65 (iv) *n* = 1137 (v) Screening: CSID and five-word test (vi) Dementia diagnosis: DSM-IV (vii) Probable and possible AD: NINCDS-ADRDA	(i) Dementia prevalence was not significantly higher in urban versus rural community: 3.7% versus 2.6% (ii) Prevalence rate of dementia in this urban community was similar to that reported in other cities in developing countries (iii) AD is the most common cause of dementia in urban setting		(i) Age and female gender associate with dementia (ii) Differences in level of education did not affect dementia prevalence

Central African Republic and Republic of Congo	Guerchet et al., **2010** [11], Guerchet et al., **2012** [37], Mbelesso et al., **2012** [12], and Guerchet et al., **2013** [38]	(i) Multicenter cross-sectional community-based surveys (ii) Conducted in 2 urban communities (Bangui and Brazzaville) (iii) Age ≥ 65 (iv) *n* = 496 in Bangui and 520 in Brazzaville (Bangui in Central African Republic and Brazzaville in Republic of Congo) (v) Screening: CSID and five-word test (vi) Dementia diagnosis: DSM-IV (vii) Probable and possible AD: NINCDS-ADRDA	**2010**: prevalence rates of dementia in these 2 urban areas of central Africa is similar to high income countries: 8.1% in Bangui and 6.7% in Brazzaville		(i) **2012**: increasing age, female gender, hypertension, a body mass index < 18.5, depressive symptoms, and the lack of a primary education were significantly associated with dementia (ii) Life events (death of one parent during childhood and recent move) were also associated with increased risk of dementia (iii) **2013**: there is a link between atherosclerosis (represented by low ankle-brachial index) and cognitive disorders in Africans as previously reported in AA and other ethnic groups

Kenya	Chen et al., **2010** [39]	(i) Setting: rural community (ii) Cross-sectional study evaluating the usefulness of Kikuyu version of the CSID in diagnosis of AD (iii) **Age** = 70–96 years (mean age 70.7) (iv) **n** = 184 (84 controls versus 100 demented subjects) (v) Diagnosis of dementia: ICD-10 and DSM-III-R (vi) APOE genotyping was conducted (vii) Blood test: for HIV and syphilis			(i) There was no association between years of education or vascular factors (diabetes, stroke, lipid levels, and hypertension) and dementia status (ii) APOE-*ε*4 allele frequencies were high (~30%) and not different between normal subjects and those with probable AD

Tanzania	Longdon et al., **2013** [40] Tanzania (six rural communities)	(i) Two-phase cross-sectional survey (ii) Age ≥ 70 years (iii) *n* = 1198 (iv) 6 rural communities (villages) (v) Screening tool: CSID (vi) Dementia diagnosis: DSM-IV	(i) Age-standardized prevalence of dementia was 6.4% (ii) Prevalence of dementia in rural Tanzanian population is similar to that reported in high income countries		(i) Dementia prevalence rates increased with increasing age (ii) Education was not a significant predictor of dementia
	Paddick et al., 2013 [6]	(i) Two-phase cross-sectional survey (ii) *Aim*: to compare prevalence rates obtained in the study by Longdon et al., 2013 [40] using the DSM-IV criteria for diagnosis of dementia with those obtained using the 10/66 diagnostic criteria, which is specifically designed for use in low and middle income countries (iii) Age ≥ 70 years (iv) *n* = 1198 (v) 6 rural communities (villages) (vi) Screening tool: CSID (vii) Dementia diagnosis: 10/66 diagnostic criteria	Prevalence of dementia was found to be 21.6% (AD prevalence not reported) in the rural Hai district of Tanzania using the 10/66 diagnostic criteria for dementia		Education was a significant predictor of “10/66 dementia,” but not of DSM-IV dementia

AA: African Americans; AD: Alzheimer's dementia; AGECAT: automated geriatric examination for computer-assisted taxonomy; CERAD: the consortium to establish a registry for Alzheimer's disease; CSID: community screening instrument for dementia; DSM-III-R: diagnostic and statistical manual of mental disorders 3rd edition revised; DSM-IV: diagnostic and statistical manual of mental disorders 4th edition; ICD-10: international classification of diseases 10th revision; GMS: the geriatric mental status schedule; MMSE: minimental state examination; SDT: stick design test; NSAID: nonsteroidal anti-inflammatory drugs; NINCDS-AIREN: National Institute of Neurological Disorders and Stroke and the *Association Internationale pour la Recherche et l'Enseignement en Neurosciences*; NINCDS-ADRDA: *National Institute of Neurological and Communicative Disorders and Stroke* and the Alzheimer's Disease and Related Disorders Association; VaD: vascular dementia.

**Table 2:** Epidemiologic research on dementia in sub-Saharan Africa—hospital-based studies.

Country	Authors	Study design/methodology	Prevalence rate	Incidence rate	Risk factors/associated conditions
Nigeria	Ogunniyi et al., **1993** [41]	(i) Cross-sectional hospital-based study conducted in Nigeria (Ibadan) (ii) Age ≥ 47 years (iii) *n* = 57,440; 37 cases had dementing illnesses (iv) Diagnostic tools: ICD-10R, NINCDS-ADRDA, and Hachinski ischemic score	(i) 37 out of 57,440 (0.064%) hospitalized patients had dementing illnesses (ii) Eighteen cases out of 37 (48.65%) had vascular dementia (iii) Probable primary degenerative dementia accounted for only one case; it was therefore postulated that it is rare in Nigeria		Hypertension, parkinsonism, diabetes, and benign prostatic hyperplasia were associated with dementia
	Osuntokun et al., **1995a** [42]	(i) Autopsy survey of brains (ii) Age ≥ 40 years (iii) *n* = 198 brains of Nigerians including 45 brains of people of age ≥ 65 years			Histological markers of AD (cortical neuronal loss, amyloid beta plaques, neurofibrillary tangles, and amyloid angiopathy) are minimum or absent
	Baiyewu et al., **1997** [43]	(i) Cross-sectional survey of 2 nursing homes in Lagos (ii) Age: elderly—age range not disclosed (iii) *n* = 23 (iv) Psychiatric disorders diagnosis: DSM-III-R and AGECAT	(i) Dementia prevalence was 47.83% (11 out of 23 patients) (ii) Prevalence of psychiatric disorders is similar to that found in similar institutions in industrialized countries		
	Ekenze et al., **2010** [44]	(i) Cross-sectional survey (2003–2007) of neurological admissions at a university hospital in South East Nigeria (ii) *n* = 1249; 51% male, 49% female	Prevalence of dementia was 3% of all neurological diagnoses		
	Amoo et al., **2011** [45]	(i) Cross-sectional survey (ii) Review of hospital records of patients with diagnoses of dementia or dementing illness at a neuropsychiatric practice in South-Western Nigeria (1998–2007) (iii) *n* = 240, 294 (iv) Diagnostic tools: ICD-10 criteria as	(i) Prevalence of probable dementia was 0.045% (ii) AD and VaD were the predominant subtypes, accounting for 57.41% and 18 16.67% of cases, respectively		

Ghana	Turkson and Asamoah, **1997** [46]	(i) Retrospective study at a psychiatric outpatient clinic in the city of Accra (ii) Age ≥ 60 years (iii) *n* = 35 (iv) Diagnostic tool: ICD-10	Dementia is the second most common psychiatric disorder among 35 patients who attended a psychiatric outpatient clinic in Accra, Ghana		

Kenya and Tanzania (East Africa)	Kalaria et al., **1997** [47]	(i) Autopsy surveys of brain tissue (ii) Histology of brains of east Africans to search for typical lesions of AD (Amyloid beta deposits and neurofibrillary tangles)			(i) Reported high frequency of APOE-*ε*4 in elderly east Africans without dementia (ii) Study suggests that APOE-*ε*4 is a nonspecific factor for dementia in east Africans
	Winkler et al., **2011** [48]	(i) Prospective analysis carried out over 8 months at a hospital in northern Tanzania (ii) *n* = 768 (iii) Subjects had neurological/psychiatric diagnoses (iv) Diagnostic tool: Glasgow Coma Scale	(i) Dementia was the cause of acute confusion in 6.9% of cases (ii) In 0.2% cases, dementia was the cause of impaired consciousness		

Senegal	Touré et al., **2008** [49, 50]	(i) Cross-sectional survey conducted from 2004-2005 (ii) Age ≥ 55 years (iii) *n* = 872 (iv) Phase 1: questionnaire (v) Phase 2: clinical exam and neuropsychiatric evaluation	Prevalence of dementia is 6.6% at a university medical center in the city of Dakar		
	Touré et al., **2012** [51]	(i) Cross-sectional survey (ii) Age ≥ 65 years (iii) *n* = 507, urban area (iv) Screening tool: “the test of Senegal” (v) Dementia diagnosis: DSM-IV-R	Prevalence of dementia estimated at 8.87% at a geriatric health center in city of Dakar		Age, illiteracy, and low social network linked to high risk of dementia in elderly as in Western countries

Burkina Faso	Napon et al., **2009** [52]	(i) Cross-sectional survey (2005 to 2007) (ii) Age ≥ 15 years (iii) *n* = 15, 815 (iv) Dementia diagnosis: DSM-IV	(i) Prevalence of dementia in patients who visited the hospital and consulted in the various services in this university hospital was 0.46% (ii) Prevalence of dementia in hospitalized patients was 2.21% (iii) AD was the most prevalent type (iv) Average age of patients diagnosed with dementia was 62.20 years		

South Africa	Kalula et al., **2010** [53]	(i) Cross-sectional survey (2003–2008) (ii) *n* = 305 (iii) University of Cape Town Memory Clinic (iv) Age: 37–89 (v) Mean age: 70	Dementia made up 74% of all diagnoses at a memory clinic		
	Ramlall et al., **2013** [54]	(i) Cross-sectional survey (ii) Conducted in group residential homes for the elderly (iii) Age ≥ 60 years (iv) *n* = 140 (v) 97 females, 43 males (vi) 106 participants had less than 12 years education (vii) Diagnosis of dementia based on DSM-IV-TR	(i) Dementia prevalence was 7.9% (ii) Prevalence of MCI was 37.1%		(i) Increasing age associated with MCI and dementia (ii) No association between gender and cognitive impairment (iii) MCI associated with lower education level (iv) Prevalence of vascular risk factors ranged from 66.4% (hypertension) to 14.3% (stroke) (v) Subjective memory complaints were significantly associated with cognitive impairment

Cameroon	Tegueu, et al., **2013** [55]	(i) Retrospective chart review over 6-year period (ii) Age ≥ median of 45 years (iii) *n* = 4526 (iv) Urban outpatient clinic (v) Diagnostic tool: ICD-10	(i) Prevalence of dementia was 2.85% at %) at an urban clinic with neurological consultation service (ii) Dementia was the tenth leading neurologic disease		

AA: African Americans; AGECATL: automated geriatric examination for computer-assisted taxonomy; CERAD: the consortium to establish a registry for Alzheimer's disease; CSID: community screening instrument for dementia; DSM-III-R: diagnostic and statistical manual of mental disorders 3rd edition revised; DSM-IV: diagnostic and statistical manual of mental disorders 4th edition; ICD-10: international classification of diseases 10th revision; GMS: the geriatric mental status schedule; MMSE: minimental state examination; SDT: stick design test; NSAID: nonsteroidal anti-inflammatory drugs; NINCDS-AIREN: National Institute of Neurological Disorders and Stroke and the *Association Internationale pour la Recherche et l'Enseignement en Neurosciences*; NINCDS-ADRDA: *National Institute of Neurological and Communicative Disorders and Stroke* and the Alzheimer's Disease and Related Disorders Association.
